# Unveiling the propagation dynamics of self-accelerating vector beams

**DOI:** 10.1038/srep34272

**Published:** 2016-09-27

**Authors:** Jonathan Bar-David, Noa Voloch-Bloch, Noa Mazurski, Uriel Levy

**Affiliations:** 1 Department of Applied Physics, The Benin School of Engineering and Computer Science, The Center for Nanoscience and Nanotechnology, The Hebrew University of Jerusalem, Jerusalem, 91904, Israel

## Abstract

We study theoretically and experimentally the varying polarization states and intensity patterns of self-accelerating vector beams. It is shown that as these beams propagate, the main intensity lobe and the polarization singularity gradually drift apart. Furthermore, the propagation dynamics can be manipulated by controlling the beams’ acceleration coefficients. We also demonstrate the self-healing dynamics of these accelerating vector beams for which sections of the vector beam are being blocked by an opaque or polarizing obstacle. Our results indicate that the self-healing process is almost insensitive for the obstacles’ polarization direction. Moreover, the spatial polarization structure also shows self- healing properties, and it is reconstructed as the beam propagates further beyond the perturbation plane. These results open various possibilities for generating, shaping and manipulating the intensity patterns and space variant polarization states of accelerating vector beams.

Airy beams, as part of the group of wave-functions coined sometimes “non-diffracting beams” or “self-accelerating beams” have drawn significant scientific interest, due to their shape-preserving and accelerating propagation dynamics in space or in time. Ever-since their first introduction^1^, Airy beams have been investigated in a wide range of physical systems^2^,^3^,^4^,^5^, in various symmetries^6^,^7^, in linear and nonlinear mediums^4^,^8^, and suggested for many applications such as micro-particles manipulations^9^, optical routing^10^ and super resolution imaging^11^. The research of Airy beams has also triggered the developing of other forms of accelerating beams^12^,^13^,^14^,^15^. One of the most intriguing properties of self-accelerating beams is their ability to self-heal from blocking obstacles^16^, a property now researched in the context of other beam types and polarizations^17^,^18^,^19^. The common way to characterize accelerating beams is by tracing the trajectory of the highest intensity lobe as it curves while propagating through space. To date, in the vast majority of reported experiments, a linearly polarized Gaussian beam has been used as the excitation source. While such beams are probably the most common form of coherent light source, we are recently witnessing a growing interest in light with a spatially varying polarization, generally known as vector beams^20^.

One of the many types of vector beams is the radially polarized mode, which exhibits a spatially varying polarization, with the transverse electric field directed outward from the optical axis, and which is an axial-symmetric solution to Maxwell’s equations^21^,^22^,^23^. Now implemented in different systems, radially polarized light was proved to be efficient in nano-focusing^24^,^25^,^26^,^27^,^28^,^29^, microscopy and particle manipulations^30^.

The combination of Airy patterns and vector beams (shown in Fig. 1), which can be defined as Airy vector beams, has been somewhat overlooked by researchers, with very little reports so far^7^,^31^. In such beams, the interplay between the Airy pattern and polarization states, symmetry differences and different propagation dynamics, rises many interesting outcomes.

In this report we present theoretical predictions along with numerical and experimental results showing the propagation dynamics, self-healing mechanisms and polarization distribution of radially-polarized vector Airy beams (RAVB). While RAVBs are the basic form of accelerating vector beams, this research can be further extended to any accelerating wave packet and any type of polarization structure.

This paper will show that when a radially polarized Gaussian beam illuminates a cubic phase mask and focused, RAVBs are generated at the focal plane. As illustrated in Fig. 1c, the main lobe of RAVB’s is divided into two lobes by a radially shaped polarization singularity (zero-order topological charge^32^). The checkerboard pattern of the RAVB tail at the focal plane is also formed by the radial polarization as every two adjacent lobes interfere destructively with anti-phase relations.

Furthermore, as the beam propagates, it will be shown that the usual form of accelerating Airy beams can be observed, with a linearly polarized main lobe appearing and accelerating in space, while the polarization singularity is broadened and drifted into the beam’s tail.

## Theoretical Background

Radially polarized light can be described as a solution of the wave-equation in cylindrical coordinate system^20^,^21^,^22^,^23^. Its transverse electric field component is given in the form.


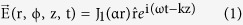


Where *J*_1_(*x*) is the first order radial Bessel function, *r* and 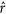 are the radial coordinate and the radial unit vector respectively, and k is the propagation wavenumber in the propagation (z) direction. The radially polarized beam has a distinct signature in its Stokes parameters, namely the four-lobed structure of S1 and S2 parameters as depicted in Fig. 2 (top row). This unique signature will be used further in this report to establish the polarization properties of radially polarized Airy beams.

The diffraction of a radially polarized beam by a grating will result in the creation of multiple diffraction orders, each of them still holds radial polarization distribution, as demonstrated previously^29^.

**Figure 1:**
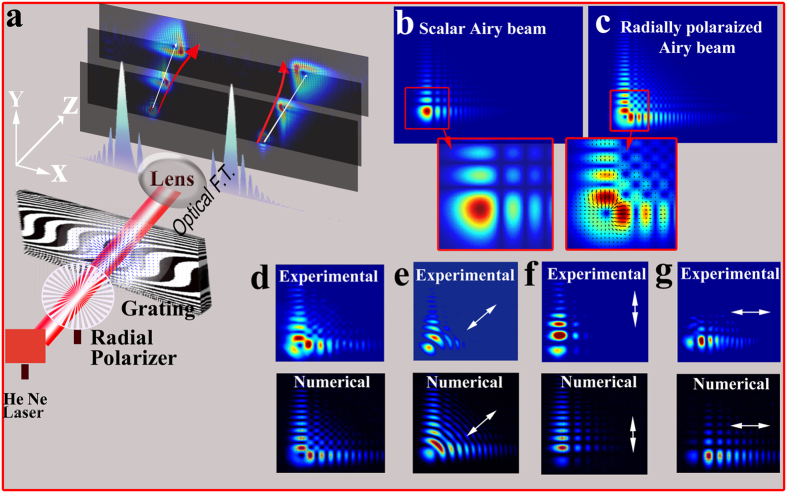
Generating radially polarized self-accelerating vector beams. (**a**) A radially polarized He-Ne laser beam is diffracted from a binary modulated cubic phase mask. Then, it is optically Fourier transformed and recorded at the focal plane and beyond. As illustrated, the radial polarization singularity (white line) and the highest intensity lobe (red line) propagate along different trajectories. (**b,c**) Comparison between the numerically calculated diffraction patterns of the known scalar Airy beam and the radially polarized Airy beam at the focal plane. As can be seen, the main intensity lobe is imprinted with radial polarization singularity. (**d–h**) Experimental and numerical results of radially polarized Airy vector beams after applying a polarizer. Polarization direction is denoted by the white arrow (un-polarized, 45° polarized, x polarized, y polarized, respectively).

An Airy beam is created by diffracting light through a binary cubic phase-grating with transmission function of the following shape: 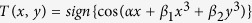
33,34. This transmission function creates the diffraction pattern presented in Fig. 1c, which is noticeably different from the linearly polarized Airy pattern presented in Fig. 1b, by the splitting of the main lobe, and the “checkerboard” pattern of the beam’s tail. These differences are direct outcomes of the polarization structure, and not of the intensity distribution alone. Under the paraxial approximation, this diffraction pattern may be analyzed by the convolution theorem^35^.


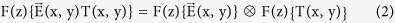


Where F(z) representsthe optical propagation by Fourier transform to plane z = f, where f is the focal length of the lens, 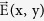 is the incident radially polarized beam and T(x, y) is the transmission function of the diffractive element generating the Airy beam. It is understood that 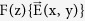 represents the diffraction of the radially polarized beam and 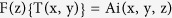 is the Airy function. While the diffraction of most beams is characterized by broadening, the Airy beam will preserve its shape, and shift laterally^1^. The convolution in Eq. 2 means that the propagation of the combined RAVB is the propagation of the two functions (Radially polarized incident beam, and Airy pattern), superimposed. Therefore, the Airy’s curved trajectory will result in a shift of the Airy pattern with respect to the origin of the radial polarization distribution as shown in Fig. 1(a). Similar separation has been observed with Airy-Vortex beams where the phase singularity is separated from the Airy’s main lobe^31^,^36^. In this paper we observed and measured for the first time, this separation dynamics for the polarization singularity and highest intensity lobe. It must be mentioned that the singularity in Vortex-Airy beams is originated from a singular phase distribution, while in our case, it evolves directly from the polarization structure, which will yield different propagation characteristics in non-isotropic media.

## Experiments Description

Our experimental setup is fairly simple (see Fig. 1a): a He-Ne laser beam (*λ* ≅ 633 nm) is passed through a radial polarization converter (Altechna) followed by a binary phase grating and a lens. The binary phase grating is fabricated on a double-polished glass substrate by standard photolithography techniques, with a transmission function defined by:


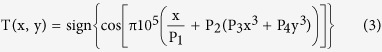


We have fabricated several gratings with different values of the parameters P_1_ to P_4_ to create Airy beams with different characteristics. Parameter P_1_ inversely controls the diffraction angle in the horizontal direction, while the combination of P_2_ along with P_3_ or P_4_ controls the Airy acceleration in the horizontal or vertical directions, respectively. The values of parameters P_1_ to P_4_ were chosen to fulfill the following requirements: (a) easy fabrication by standard photolithography procedures, which dictates that feature size is kept above 2 μm, and is also beneficial as Airy beams of high spatial frequency tend to smear over short propagation distances; (b) clear detection of all Airy lobes by our camera (i.e. separation between Airy lobes at the focal plane must be significantly larger than pixel size); and (c) the full diffraction pattern must fit into the CCD area. Typical numbers for these parameters (considering the use of standard lenses with focal lengths of 5–20 cm) are 1 < P_1_ < 10, 1000 < P_2_ < 10^5^, 1 < P_3_,P_4_ < 10, where the lateral coordinates x and y are measured in meters. The camera used to detect the Airy pattern was a sCMOS camera (Hamamatsu Orca Flash 4 V2), which is mounted on a rail to allow capturing the Airy beam at the focal plane and beyond, without losing alignment.

Stokes measurements at various distances were made by a rail-mounted set of a linear polarizer and a quarter wave plate.

## Experimental Results

**Figure 2:**
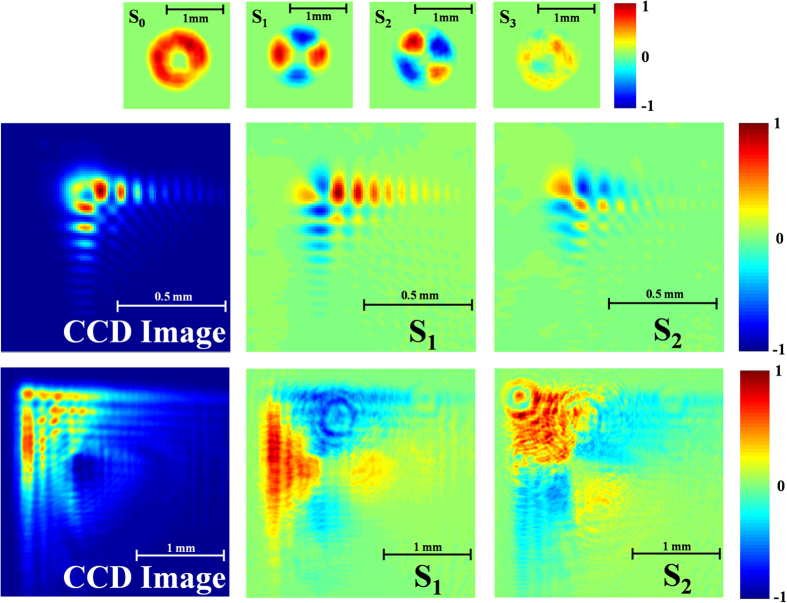
(Top row) Stokes parameters of radially polarized light. The distinct polarization structure is manifested by the four-lobe form of parameters S_1_ and S_2_. S_3_ which should be zero is indeed noticeably weaker yet it is not identically zero due to inaccuracies in polarizers and QWPs used. (Middle row) CCD image and S_1_ and S_2_ parameters for RAVB’s at the focal plane. Note the clear sign of radial polarization. (Bottom row) S_0_–S_2_ parameters for the RAVB after propagating 10 cm beyond the focal plane. As can be observed, the radial polarization singularity is drifted towards the beam’s tail.

**Figure 3:**
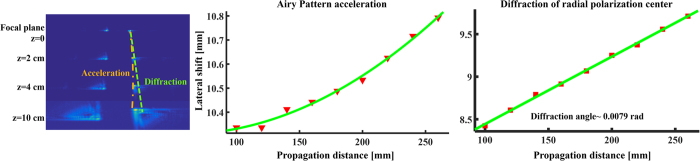
Propagation analysis of radially polarized Airy beams. The Airy pattern is examined in various distances from the focal plane. The rightmost panel shows the experimentally measured intensity patterns at different propagation distances, while the middle and left panels analyze the propagation and verify the theoretical prediction of acceleration and diffraction.

**Figure 4:**
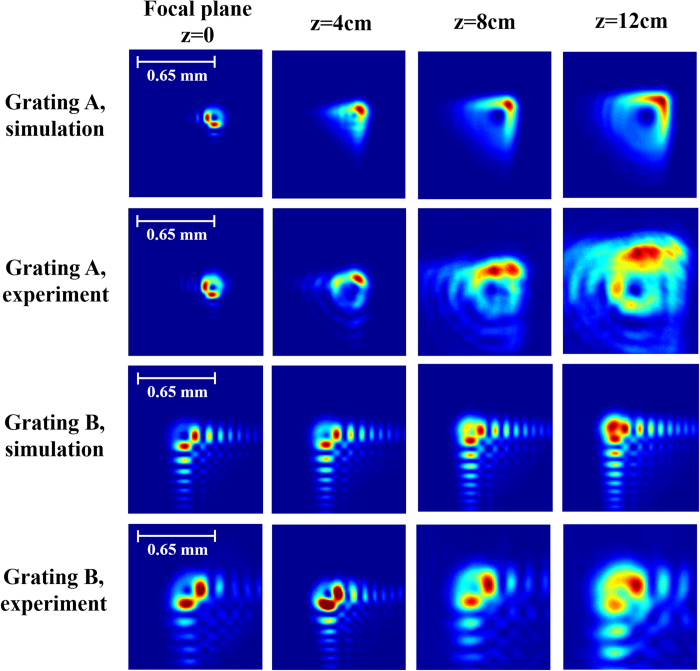
Shaping vector beams’ dynamics by acceleration. Two top rows show calculated and experimental intensity patterns for high-acceleration Airy patterns at focal plane and in 4 cm steps beyond it. The Airy grating coefficients (as described in Eq. 3) used were P_1_ = 4, P_2_ = 5000, P_3_ = P_4_ = 1. The two bottom rows show the calculated and experimental patterns for radially polarized Airy Beams with acceleration 40 times smaller (compared to top rows), with grating coefficients P_1_ = 4, P_2_ = 20000, P_3_ = P_4_ = 1. It is easily seen that the separation rate of radial polarization center from Airy pattern is determined by the Airy acceleration coefficients. The faster broadening of experimental data is caused by non-perfect beam collimation.

**Figure 5:**
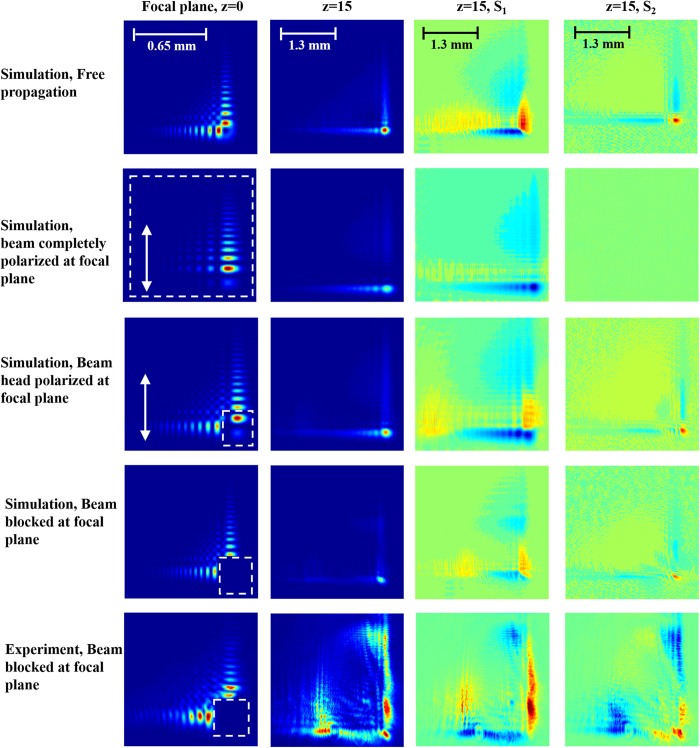
Polarization reconstruction of an RAVB diffracted by an obstacle at the focal plane. In all focal plane panels, the perturbation is denoted by a white, dashed line square, and the polarized transmission axis is denoted by a white arrow. Top row shows the propagation of a radially polarized Airy beam without perturbation is calculated (compare with Fig. 4). 2^nd^ row describes the case where a uniform linear polarizer is applied to the beam at the focal plane. As can be seen, the beam resembles a linearly polarized Airy beam in shape and propagation. Middle row describes the case of a polarization dependent obstacle which is applied at the focal plane, and beam propagation is calculated. From the results it is clear that such an obstacle barely changes the beam’s propagation characteristics, as the propagating beam resembles the non-perturbed case presented in the topmost row. 4^th^ row and bottom row present, the calculated and measured beam propagation beyond an opaque obstacle at the focal plane, respectively. The Stokes parameters of the propagated beam consist of the four-lobe structure typical of radial polarization, which is an indication for polarization reconstruction.

Our experimental results are presented in Figs 2, 3, 4 and 5. Figure 2 presents the intensity distribution and Stokes parameters S_1_ and S_2_ of the RAVB at the focal plane and 10 cm beyond the focal plane. We observe experimentally the splitting of the main lobe and the checkerboard pattern of beams’ tail. From observing the Stokes parameters, we notice that the radial polarization structure is indeed imprinted in all the Airy lobes, and the checkers-board structure is the result of destructive interference of anti-phase fields of adjacent lobes. Next, we measured the Stokes parameters of a beam propagating beyond the focal plane, presented in Fig. 2. From these measurements we learn that the change in the beam’s shape is the result of the gradual separation between the radial polarization structure and the Airy pattern. This understanding allows us to study the propagation dynamics of the intensity and polarization of these beams, presented in Figs 3, 4 and 5. As shown in Fig. 3, the radial polarization center is diffracted at the angle expected according to the grating periodicity, whereas the Airy lobes are accelerated and we notice the predicted separation between these structures, i.e. the polarization singularity of the radial beam drifts away from the main lobe towards the RAVB’s tail. This effect is much more pronounced when the acceleration is large, as depicted in Fig. 4, which compares numerically and experimentally between similar beams with a factor of 40 in their acceleration constant. It is seen that for high-acceleration beam (beam A in Fig. 4’s top panels) the separation of the radial polarization and Airy pattern occurs over much shorter distances. Finally, self-healing experiments were made to fully understand the propagation characteristics of RAVBs. These results, alongside with numerical calculations are shown in Fig. 5.

The self-healing experiments were done by introducing a perturbation to the beam at the focal plane and examining the propagation of the beam as described previously.

We have considered two types of perturbations: A - polarizing the main lobe, B - completely blocking the main lobe. Both cases are presented in Fig. 5. The first case was studied numerically, while the latter was studied both numerically and experimentally.

The first row of Fig. 5 shows the propagation of the non-perturbed RAVB. This will be used as a reference for the following cases. The second row of Fig. 5 shows the RAVB after applying a uniform linear polarizer in the direction of the white arrow. Here, the radial pattern is essentially lost and the beam resembles the more conventional linearly polarized Airy beam.

When polarizing the main lobe alone, as described in the middle row of Fig. 5, we only introduce a small perturbation to the beam, and the reconstructed pattern is nearly indistinguishable comparing with the unperturbed case (top row).

The last two rows (numerical calculation and experimental measurements, respectively), describe the case in which the main lobe of the beam is completely blocked. As can be seen, in spite of the significant perturbation, the RAVB shows fantastic polarization self- healing characteristics, as evident by the stokes parameters in which the four-lobed pattern can be observed after propagating 15 centimeters away from the focal plane. This result can be explained by the Fourier-optics approach: as the main lobe is blocked in the Fourier plane, the beam will maintain its spatial structure, but will suffer a decrease in contrast. This is because each point along the reconstruction plane is being generated by coherent contributions from all points along the beam at the obstacle plane. The polarization distribution is thus only weakly affected by the blocking, as the other lobs maintain their polarization structure and contribute to the vector field reconstruction as the beam propagates away from the obstacle. We can thus conclude that the information of the local radial polarization singularity is carried in a nonlocal manner by the entire wave-front, i.e., the vectorial propagation dynamics is not described by a local vectorial variation dynamics near the main lobe, but instead by the vectorial states of the light which emerges from the additional sidelobes^37^.

## Conclusions

We have reported on the propagation and self-healing dynamics of Airy vector beams. These radially polarized Airy beams belong to the broader family of accelerating vector beams. While such beams are rarely considered, they potentially hold many intriguing phenomena. Our results show that for a freely-propagating beam, the acceleration of the Airy beam has a major role in the evolution of the beam’s polarization state.

For RAVB passing an obstacle, the self-healing process is polarization insensitive - a single reconstructed main lobe appears in all cases examined, with polarization properties similar to those of a freely-propagating beam.

The polarization reconstruction of the self-healed RAVB is an important result. The RAVB feature which enables this reconstruction is the singularity data imprinted in the Airy beam’s tail. The reconstruction therefore shows that the airy beam’s tail carries the full information (amplitude, phase and polarization) needed to reconstruct the beam, and not the information of intensity alone^37^.

The polarization of light is a degree of freedom often overlooked, but can be of great significance in many fields such as beam-shaping and micro-particle manipulations. It is our hope that this work will be followed by additional demonstrations to provide further advancements in the field. For example, one may consider generating arbitrary accelerating wave packets with even more complex polarizations structures. Furthermore, the propagation dynamics of these beams can be investigated inside birefringent media, for which each polarization component may have a different phase velocity. One may also consider the case where the beam trajectory goes beyond the scalar approximation. Finally, it might be of interest to test the self-focusing properties of these beams inside Kerr type nonlinear medium, which can also affect the polarization and intensity patterns of the RAVBs.

## Additional Information

**How to cite this article**: Bar-David, J. *et al.* Unveiling the propagation dynamics of self-accelerating vector beams. *Sci. Rep.* **6**, 34272; doi: 10.1038/srep34272 (2016).

## References

[b1] Siviloglou G. A., Broky J., Dogariu A. & Christodoulides D. N. Observation of Accelerating Airy Beams. Phys. Rev. Lett. 99, 213901 (2007). 18233219 10.1103/PhysRevLett.99.213901

[b2] Voloch-Bloch N., Lereah Y., Lilach Y., Gover A. & Arie A. Generation of electron Airy beams. Nature 494, 331–335 (2013). 23426323 10.1038/nature11840

[b3] Polynkin P., Kolesik M., Moloney J. V., Siviloglou G. A. & Christodoulides D. N. Curved Plasma Channel Generation Using Ultraintense Airy Beams. Science 324, 229–232 (2009). 19359582 10.1126/science.1169544

[b4] Bekenstein R., Schley R., Mutzafi M., Rotschild C. & Segev M. Optical simulations of gravitational effects in the Newton-Schrodinger system. Nat Phys 11, 872–878 (2015).

[b5] Fu S., Tsur Y., Zhou J., Shemer L. & Arie A. Propagation Dynamics of Airy Water-Wave Pulses. Phys. Rev. Lett. 115, 34501 (2015). 10.1103/PhysRevLett.115.034501 26230797

[b6] Vaveliuk P., Lencina A., Rodrigo J. A. & Matos O. M. Symmetric Airy beams. Opt. Lett. OL 39, 2370–2373 (2014). 10.1364/OL.39.002370 24978995

[b7] Zhang P. *et al.* Trapping and guiding microparticles with morphing autofocusing Airy beams. Opt. Lett. OL 36, 2883–2885 (2011). 10.1364/OL.36.002883 21808346

[b8] Ellenbogen T., Voloch-Bloch N., Ganany-Padowicz A. & Arie A. Nonlinear generation and manipulation of Airy beams. Nat Photon 3, 395–398 (2009).

[b9] Baumgartl J., Mazilu M. & Dholakia K. Optically mediated particle clearing using Airy wavepackets. Nat Photon 2, 675–678 (2008).

[b10] Rose P., Diebel F., Boguslawski M. & Denz C. Airy beam induced optical routing. Applied Physics Letters 102, 101101 (2013).

[b11] Jia S., Vaughan J. C. & Zhuang X. Isotropic three-dimensional super-resolution imaging with a self-bending point spread function. Nat Photon 8, 302–306 (2014). 10.1038/nphoton.2014.13 PMC4224117 25383090

[b12] Kaminer I., Bekenstein R., Nemirovsky J. & Segev M. Nondiffracting Accelerating Wave Packets of Maxwell’s Equations. Phys. Rev. Lett. 108, 163901 (2012). 22680719 10.1103/PhysRevLett.108.163901

[b13] Greenfield E., Segev M., Walasik W. & Raz O. Accelerating Light Beams along Arbitrary Convex Trajectories. Phys. Rev. Lett. 106, 213902 (2011). 21699298 10.1103/PhysRevLett.106.213902

[b14] Alpmann C., Bowman R., Woerdemann M., Padgett M. & Denz C. Mathieu beams as versatile light moulds for 3D micro particle assemblies. Optics Express 18, 26084 (2010). 21164957 10.1364/OE.18.026084

[b15] Bandres M. A. & Rodríguez-Lara B. M. Nondiffracting accelerating waves: Weber waves and parabolic momentum. New J. Phys. 15, 13054 (2013).

[b16] Broky J., Siviloglou G. A., Dogariu A. & Christodoulides D. N. Self-healing properties of optical Airy beams. Opt. Express, OE 16, 12880–12891 (2008). 10.1364/oe.16.012880 18711527

[b17] Milione G. *et al.* Measuring the self-healing of the spatially inhomogeneous states of polarization of vector Bessel beams. J. Opt. 17, 35617 (2015).

[b18] Vyas S., Kozawa Y. & Sato S. Self-healing of tightly focused scalar and vector Bessel–Gauss beams at the focal plane. J. Opt. Soc. Am. A JOSAA 28, 837–843 (2011). 10.1364/JOSAA.28.000837 21532695

[b19] McLaren M., Mhlanga T., Padgett M. J., Roux F. S. & Forbes A. Self-healing of quantum entanglement after an obstruction. Nature Communications 5, 3248 (2014). 10.1038/ncomms4248 24500069

[b20] Zhan Q. Cylindrical vector beams: from mathematical concepts to applications. Adv. Opt. Photon. AOP 1, 1–57 (2009).

[b21] Wolf E. Electromagnetic Diffraction in Optical Systems. I. An Integral Representation of the Image Field. Proceedings of the Royal Society of London A: Mathematical, Physical and Engineering Sciences 253, 349–357 (1959).

[b22] Richards B. & Wolf E. Electromagnetic Diffraction in Optical Systems. II. Structure of the Image Field in an Aplanatic System. Proceedings of the Royal Society of London A: Mathematical, Physical and Engineering Sciences 253, 358–379 (1959).

[b23] Schimpf D. N., Putnam W. P., Grogan M. D. W., Ramachandran S. & Kärtner F. X. Radially polarized Bessel-Gauss beams: decentered Gaussian beam analysis and experimental verification. Opt. Express OE 21, 18469–18483 (2013). 10.1364/OE.21.018469 23938719

[b24] Quabis S., Dorn R., Eberler M., Glöckl O. & Leuchs G. Focusing light to a tighter spot1. Optics Communications 179, 1–7 (2000).

[b25] Dorn R., Quabis S. & Leuchs G. Sharper Focus for a Radially Polarized Light Beam. Phys. Rev. Lett. 91, 233901 (2003). 14683185 10.1103/PhysRevLett.91.233901

[b26] Lerman G. M., Yanai A. & Levy U. Demonstration of Nanofocusing by the use of Plasmonic Lens Illuminated with Radially Polarized Light. Nano Lett. 9, 2139–2143 (2009). 19391611 10.1021/nl900694r

[b27] Sheppard C. J. R. & Choudhury A. Annular pupils, radial polarization, and superresolution. Appl. Opt. AO 43, 4322–4327 (2004). 10.1364/ao.43.004322 15298403

[b28] Yanai A. & Levy U. Plasmonic focusing with a coaxial structure illuminated by radially polarized light. Opt. Express OE 17, 924–932 (2009). 10.1364/oe.17.000924 19158907

[b29] Bar-David J., Lerman G. M., Stern L., Mazurski N. & Levy U. Generation of a periodic array of radially polarized Plasmonic focal spots. Opt. Express 21, 3746–3755 (2013). 23481831 10.1364/OE.21.003746

[b30] Zhan Q. Trapping metallic Rayleigh particles with radial polarization. Opt. Express OE 12, 3377–3382 (2004). 10.1364/opex.12.003377 19483862

[b31] Zhou J., Liu Y., Ke Y., Luo H. & Wen S. Generation of Airy vortex and Airy vector beams based on the modulation of dynamic and geometric phases. Opt. Lett. OL 40, 3193–3196 (2015). 10.1364/OL.40.003193 26125400

[b32] Allen L., Beijersbergen M. W., Spreeuw R. J. C. & Woerdman J. P. Orbital angular momentum of light and the transformation of Laguerre-Gaussian laser modes. Phys. Rev. A 45, 8185–8189 (1992). 9906912 10.1103/physreva.45.8185

[b33] Dai H. T., Sun X. W., Luo D. & Liu Y. J. Airy beams generated by a binary phase element made of polymer-dispersed liquid crystals. Opt. Express OE 17, 19365–19370 (2009). 10.1364/OE.17.019365 19997157

[b34] Lee W.-H. Binary computer-generated holograms. Appl. Opt., AO 18, 3661–3669 (1979). 10.1364/AO.18.003661 20216666

[b35] Goodman J. W. Introduction to Fourier Optics. (Roberts and Company Publishers, 2005).

[b36] Dai H. T., Liu Y. J., Luo D. & Sun X. W. Propagation dynamics of an optical vortex imposed on an Airy beam. Opt. Lett. OL. 35, 4075–4077 (2010). 10.1364/OL.35.004075 21124617

[b37] Kaganovsky Y. & Heyman E. Wave analysis of Airy beams. Optics Express 18, 8440 (2010). 20588690 10.1364/OE.18.008440

